# Supplementary material for: Surfing the Waves: Differences in Hospitalised COVID-19 Patients across 4 Variant Waves in a Belgian University Hospital
Source: Viruses. 2023 Feb 23;15(3):618. doi: 10.3390/v15030618 (PMC10057609; doi:10.3390/v15030618)
Supplement: Supplementary file 1 [file viruses-15-00618-s001.zip › viruses-2212963-supplementary.pdf]

Table S1. Additional demographic data.

Period of Admission	Pre-VOC	Alpha	Delta	Omicron	p-Value
Number of patients (% within wave)	**814**	**174**	**265**	**73**	na
**Ethnicity**					<0.001
Caucasian	429 (55.9%)	82 (47.7%)	129 (49.2%)	45 (65.2%)	
Sub-saharan African	65 (8.5%)	8 (4.7%)	18 (6.9%)	3 (4.3%)	
North-African	131 (17.1%)	59 (34.3%)	58 (32.4%)	17 (24.6%)	
Asian / Oriental	7 (0.9%)	3 (1.7%)	4 (1.5%)	2 (2.9%)	
Indian subcontinent	3 (0.4%)	5 (2.9%)	0 (0.0%)	0 (0.0%)	
South-American	2 (0.3%)	0 (0.0%)	1 (0.4%)	0 (0.0%)	
Multiracial	0 (0.0%)	1 (0.6%)	0 (0.0%)	0 (0.0%)	
Do not know	123 (16.0%)	14 (8.1%)	21 (8.0%)	1 (1.4%)	
Other	7 (0.9%)	0 (0.0%)	4 (1.5%)	1 (1.4%)	
**BMI**	28.2 ±5.7	28.9 ±5.5	28.5 ±6.1	27.7 ±6.2	0.084
Normal weight (< 24,9)	245 (32.0%)	40 (23.1%)	81 (31.0%)	26 (37.1%)	0.177
Overweight (25 – 29,9)	273 (35.6%)	70 (40.5%)	87 (33.3%)	24 (34.3%)	
Obesity class I (30-34,9)	147 (19.2%)	40 (23.1%)	67 (25.7%)	12 (17.1%)	
Obesity class II (>35)	101 (13.2%)	23 (13.3%)	26 (10.0%)	8 (11.4%)	
